# Testosterone Retention Mechanism in Sertoli Cells: A Biochemical Perspective

**DOI:** 10.2174/1874091X01812010103

**Published:** 2018-06-29

**Authors:** Manjeet Kaur Gill-Sharma

**Affiliations:** Neuroendocrinology Department (retired), National Institute for Research in Reproductive Health (ICMR), J. M. Street, Parel, Mumbai, 400012, India

**Keywords:** Testosterone, Androgen-binding protein, Sex hormone-binding globulin, Megalin, Sertoli cell, Spermatogenesis

## Abstract

Mechanism(s) involved in regulating Intratesticular Testosterone levels (iT) have assumed importance in recent years, from the point of view of hormonal contraception. Contraceptives using Testosterone (T) in combination with Progestins (P), for more effective suppression of pituitary gonadotropins thereby iT, are not 100% effective in suppressing spermatogenesis in human males, likely due to pesrsistence of Intratesticular Dihydrotestosterone (iD) in poor-responders. Several lacunae pertaining to the mechanism of action of principal male hormone T during spermatogenesis remain to be resolved. Notably, the mechanism through which T brings about the stage-specific differentiation of germ cells lacking Androgen Receptors (AR). Testosterone is a highly anabolic steroid with a rapid tissue clearance rate. T is intratesticular substrate for synthesis of Dihydrotestosterone (DHT) and Estradiol (E2) involved in spermtaogenesis. Therefore, it is important to delineate the mechanism(s) for retention of iT, in order to understand regulation of its bioavailability in testis. In depth studies, pertaining to the role of androgen-binding protein(s) in sequestration, retention and bioavailability of T/DHT are required to understand male fertility regulation. The appropriate approach to overcome this lacuna would be development of mice lacking functional testicular Androgen-Binding Protein (ABPKO), but not deficient T/DHT, Luteinizing Hormone (LH) and Follicle-Stimulating Hormone (FSH), in order to understand its physiological functions. Insights gained about androgen retention mechanism(s) from the ABPKO murine model will be of immense help in improving the efficacy of male hormonal contraceptives and infertility management.

## 1 INTRODUCTION

Testosterone (T) is a lipophilic steroidal molecule synthesized in the interstitial cells of Leydig in the testis. Androgen-Binding Protein (ABP)/SHBG sequester and solubilize serum T to facilitate transport and iT bioavailability [1]. Most mammalian species express a testicular protein that specifically binds androgens T/DHT (dihydrotestosterone) with high affinity [2]. The mechanism for storage of intratesticular testosterone (iT), at a level several folds higher than that in circulation, however, awaits delineation. The rapidity with which the sequestered androgens dissociate from ABP suggests that it could be regulating T/DHT bioavailability at testicular Androgen Receptors (AR) [3]. This hypothesis, however, awaits experimental substantiation.

### 1.1 Role of Testosterone (T) in Male Fertility Regulation

Testosterone Radioimmunoassays (RIA) demonstrated that a plasmatic circadian T acrophase occurred in human subjects between 1-5h [4]. The crucial role of T in spermatogenesis became evident in rats treated with AR blocker, Cyproterone Acetate (CPA). Evaluation of sperm chromatin structure by flow cytometry demonstrated that Sertoli cell AR blockade prevented initiation of chromatin condensation in elongating spermatids. AR blockade reduced the fertility of male rats due to the production of poor quality epididymal spermatozoa, deficient in thiols and protamine1 [5]. Testosterone RIAs demonstrated that tamoxifen treatment reduced the levels of intratesticular androgens in adult male rat concomitant with a reduction in their siring ability [6]. Testosterone can also act to regulate spermatogenesis *via* its non-aromatizable metabolite, Dihydrotestosterone (DHT) and estradiol (E2), its aromatizable metabolite [7, 8]. Plasmatic hormone RIAs also demonstrated that a circadian E2 acrophase occurred in human subjects between 13-18h [4]. The role of E2 in fertility regulation became evident from several studies. Estrogen Receptor (ESR1) gene null mutation led to sterility in mice [9]. Steroid hormone RIAs demonstrated that high intratesticular E2 (iE) levels produced in E2-treated rats reduced intratesticular androgens, disrupted the formation of Tubulobulbar Complexes (TBCs) and led to spermiation failure. This study revealed plasmatic E2 uptake by the Sertoli cells [10, 11]. Histology of testes of estradiol-treated rats revealed a reduction in the height of Sertoli cells, attributed to lack of polymerization of cytoskeletal protein Vimentin [12, 13]. Confocal Microscopy subsequently confirmed disorganization of Sertoli cell Vimentin in E2-treated rat testis [14]. Stimulation of ESR1 and ESR2 with specific agonists PPT (4, 40, 4”-(4- Propyl-[1H] pyrazole-1, 3, 5-triyl) and DPN (2, 3-bis (4-hydroxyphenyl)-propionitrile) respectively, reduced the fertility of male rats [15]. ESR1 agonist reduced the sperm counts, evaluated by flow cytometry of testicular cells, through suppression of plasmatic gonadotropins and testosterone. Reduced T levels led to arrest of conversion of round to elongating spermatids, owing to downregulation of chromatin condensation proteins. ESR2 agonist reduced sperm counts through germ cell apoptosis, evaluated by TUNEL assay and caused spermiation failure [16]. It is tempting to suggest that E2 could be playing an autoregulatory physiological role in the predominantly androgen-dependent biological process of spermatogenesis.

### 1.2 Significance of High Concentration of Intratesticular Testosterone (IT)

Intratesticular T is the most decisive hormone for maintenance of qualitative spermatogenesis in mammals [17]. Natural and genetically engineered mutant mice have contributed to the delineation of T- dependent stages of spermatogenesis. Congenital deficiency of T in hpg mice blocked the first meiotic division and arrested spermatogenesis at pre-meiotic spermatocyte stage, reversible with T implants [18]. The significant finding that emerged from T supplementation studies was that Sertoli cells internalized steroidal molecules from the peripheral circulation. Genetic mutant studies suggested the involvement of Sertoli cell AR in mediating T effect on round spermatid adhesion and development. The arrest of spermatogenesis at pachytene stage in Androgen Receptor Knockout (ArKO) mice indicated the role of AR in adhesion of round spermatids to Sertoli cells [19]. The absence of elongating spermatids in testis of SCARKO mice unequivocally implicated Sertoli cell AR in spermiogenesis [20].

Sertoli cell iT has been implicated in the expression of adhesion-related genes namely, Rhox5, N-cadherin, connexin-43, gelsolin, laminin-γ3, occludin, testin, nectin, zyxin and vinculin [21, 22]. Androgen Response Elements (ARE) were demonstrated by chromatin immunoprecipitation in the promoters of Sertoli cell genes, namely phosphatidylinositol binding clathrin assembly protein, early endosomal autoantigen1 and syntaxin, in the testis of estrogen-treated rats [23]. The adverse effect of blocking Sertoli cell AR on germ cell genes namely Protamine1, histone deacetylase1, ubiquitin ligating enzyme, 20S proteasome α1, 5-methyl cap binding RNA-binding protein, ubiquitin-activating and conjugating enzymes, chromodomain Y-like protein, bromodomain testis-specific protein, histone deacetylase 6, histones h2b and h3, was demonstrated by RT-PCR [24]. Thus, high levels of iT are essential for mediating its molecular effects *via* Sertoli cell AR.

### 1.3 Significance of Intratesticular Testosterone (IT) Storage Mechanism to Spermatogenesis

Several studies suggested that a functional relationship exists between iT levels in Sertoli cells and differentiation of spermatozoa. CHIP (chromatin immunoprecipitation) assay demonstrated that T and its metabolites regulated testicular genes involved in actin remodeling and endocytosis, in the testis of E2 treated rats. Liganded to AR and ER beta-receptors, T and E2 recruited coregulators NcoRI, Src1 to AREs and EREs in the promoters of Picalm, Eea1, Stx5a and Arpc1b, Evl testicular genes, respectively. The presence of a storage protein could be crucial for ensuring T bioavailability for gene transcription during spermatogenesis. Testosterone and its metabolites, liganded to AR and ER beta-receptors, recruited coregulators NcoRI, Src1 to AREs and EREs in the promoters of Picalm, Eea1, Stx5a and Arpc1b, Evl testicular genes, respectively as [25]. Histological and Confocal Microscopic evaluation of the testis of E2-treated rats revealed that reduction in iT levels had affected organization of Sertoli cell cytoskeletal Vimentin [12-14]. Flow cytometric evaluation of monobromobimane (mBBr) fluorescent dye uptake by epididymal sperm, taken from CPA- and E2-treated rats, indicated a reduction in sperm thiols, thus an altered oxidation status [5, 26]. Immunoblotting studies of E2-treated rat testis also revealed reduced levels of CREMτ (cyclic AMP response element modulator), transition proteins and protamine [26].

The occurrence of plasmatic T internalization emerged from studies of T (0.3cm) and E (0.4cm) implanted (TE) rats. TE implants suppressed the cytodifferentiation of stage VII and VIII round spermatids to 16% of controls within eleven weeks. T (24cm) implants restored the arrested cytodifferentiation within four days, ostensibly by internalizing T from peripheral circulation [7]. Failure of this restorative effect to occur in the presence of either flutamide (AR Antagonist) or L685, 273 (5alpha-reductase inhibitor) revealed the significance of bioavailability of intratesticular DHT (iD) [8]. Several studies indicated the existence of an FSH-dependent mechanism that modulates androgen responsiveness of Sertoli cells [27-29]. Supplementation of gonadotropin-deficient (hpg) mutant mice with recombinant FSH (rhFSH) and T implants (0.125-1cm), followed by stereological evaluation of mutant testis revealed that FSH mediated the proliferation of pre-meiotic spermatogenic cells, ostensibly by generating the mitogenic hormone iE from iT stores [30, 31]. Immunohistochemical localization of Bromodeoxyuridine (BrdU) in spermatogonial DNA of E2-treated rat testis had demonstrated its mitotic role [12]. Steorological evaluation of testis of LurKO, ARKO and SCARKO genetic mutant mice with disrupted androgen signalling indicated that FSH could maintain spermatogonial population [19, 20, 32]. FSH apparently produced a mitotic effect on spermatogonia *via* aromatization of iT to iE. Real-time PCR studies in rats treated with specific agonists of estrogen receptors demonstrated a direct testicular role of iE. These studies detected reductions in transcripts of transition proteins, protamine1, Arpc1b, Evl, Picalm, Bcl2, Bclw, cyclin A1 and B1spermatidal genes in adult male rats with specific agonists of ESRI/2 (PPT and DPN). These studies confirmed the role of iE *via* testicular estrogen receptors ESR1 and ESR2 [16, 33].

Therefore, maintenance of high iT levels is necessary for the synthesis of iD and iE required for efficient spermatogenesis. A mechanism for storage of T in the testis would be of physiological relevance due to its lipophilic nature, high tissue clearance rates and circadian secretion. Most importantly, the circadian peak of T necessitates a testicular mechanism of retention and storage, in order to meet the physiological need of spermatogenesis for iD/iE.

### 1.4 Mechanistic Role of Androgen-Binding Protein(s) in Testosterone Retention

Sub-human mammals express a specific androgen-binding protein (ABP) of hepatic and testicular origin, besides a non-specific albumin carrier protein for plasmatic T [2]. Homo sapiens express an identical plasmatic Sex-Hormone-Binding Globulin (SHBG) of hepatic origin [1]. Albumin and androgen-binding proteins present in Systemic Circulation sequester T. ABP and SHBG are high-affinity, androgen-binding proteins, expressed from a conserved shbg gene, in a tissue-specific manner, in human and sub-human mammals, respectively. Sertoli cells secrete ABP bidirectionally into serum and seminiferous tubular fluid in rats, regulated by FSH [3, 34, 35]. Since human SHBG is of hepatic origin, the underlying reason for CREM-induced expression of a steroid-binding shbg transcript, in the acrosomes of human spermatids, is not comprehensible [36]. Radioimmunoassays detected ten-fold higher iT as compared to plasmatic T. This feature, common to all mammals, is suggestive of the existence of a common physiological mechanism for iT retention and storage in Sertoli cells. However, non-expression of human SHBG in Sertoli cells defies this logic for gaining access to ARs. SHBG is purported to mediate plasmatic T signals *via* alternative routes [37]. Megalin is a transmembrane receptor involved in uptake of sex steroids in tissues. Megalin deficiency was immunohistochemically confirmed in the testis of megalin null mice. Megalin null mice present with cryptorchidism. Male Megalin null mutant mice have reduced expression of several androgen inducible genes namely, Tex12, Morc, Stk25, Ramp2 and increased expression of androgen-repressed genes namely, Mpo, Igfbp5 [38]. SHBG can bind and transport plasmatic T into sex-steroid dependent tissues *via* Megalin receptors [39]. SHBG can also transduce plasmatic T signalling by binding to specific, non-genomic SHBG receptors expressed on the plasma membrane [40, 41].

Transgenic mice overexpressing ABP in Sertoli cells, expressed the protein from 5.5 Kb genomic DNA regions, comprising coding and 1.5 Kb regions upstream of transcription start site of rat ABP/SHBG gene [42, 43]. ABP overexpression, however, led to upregulation of aromatase and ESR2 in germ cells. Histological assessment of ABP h transgenic mouse testis revealed apoptosis of germ cells arrested at meiotic stage. These pathophysiological effects are characteristic of E2 exposure, seen in rats treated with specific ESR1 and ESR2 receptor agonists [15, 33, 44, 45]. The phenotype of ABP transgenic mouse model overlaps with those of PPT/DPN-treated rat models. Both iT and iE upregulated ABP levels in Sertoli cells of rat testis. ABP transcripts were downregulated in CPA-treated rat testis, ostensibly by accelerating autophagic clearance [5, 46]. ABP transcripts were also upregulated in E2-treated rat testis albeit downregulated in tamoxifen-(estrogen receptor antagonist) treated rat testis [26, 47]. These studies suggest an autoregulatory role of iT and iE in iT retention and regulation of bioavailability for spermatogenesis. Therefore, the role of ABP in iT storage/retention needs to be demonstrated in the ABP gene knock out mouse model.

## 2 PUTATIVE STRATEGY TO STUDY ROLE OF INTRATESTICULAR TESTOSTERONE (IT) SEQUESTRATION MECHANISM

Gene ablation would be a far better approach to study functions of proteins that upregulate target genes. Blocking Sertoli cell ARs with CPA is the pharmacological equivalent of AR gene ablation. The observed downregulation of the transcripts of several testicular genes, involved in the process of spermatid chromatin condensation during spermiogenesis, in CPA-treated rats, supports this logic [24]. Indeed, the observed upregulation of ESR1 and aromatase, concomitant with meiotic arrest and germ cell apoptosis in testis of ABP transgenic mice were pathophysiological estrogenic effects of ABP overexpression [44, 45, 48]. ABP transgenic mice presented with a phenotype that overlapped with those observed after pharmacological stimulation of germ cell ESR1/2 in rats [15, 16, 33, 44, 45, 48]. Ostensibly, gene overexpression approach failed to demonstrate the physiological role of ABP in iT retention and storage. Development of mice lacking androgen-binding protein would be the ideal approach to study its role in iT retention and storage [3]. However, in order to overcome the potential developmental problems of *via*bility and cryptorchidism, a conditional knockout of Sertoli cell shbg gene would be an appropriate approach to study the role of ABP in iT retention and spermatogenesis.

### 2.1 Structure of Murine Androgen-Binding Protein

Sex hormone-binding globulin (shbg) gene located on chromosome 11 encodes murine ABP in CDI mouse testis [49]. The 3Kb coding region expresses a 1.7Kb transcript that encodes a 4.4539Kd precursor protein, comprising 403 amino acids in eight exons. Cleavage of a signal peptide from the N-terminus of the precursor protein generates a mature 4.1183Kd protein comprising 373 residues [35]. Photoaffinity labeling identified the steroid-binding region in residues 141-150 in rat protein [50]. Expression of human SHBG deletion mutants in E coli identified amino acid residues 18-177 to be involved in steroid binding [2, 51, 52]. Two promoters regulate tissue-specific expression of murine ABP [53, 54]. P1 promoter expresses the protein from exons 1-8 in the testis. An alternative promoter upstream of P1 expresses hepatic and cerebral ABP [35, 55, 56]. The androgen-binding protein essentially exists as a dimer comprising differentially glycosylated protomers [57]. ABP/SHBG has three conserved glycosylation sites, two of Asn at the carboxyl-terminus and one of O-glycosylation in the amino-terminus [58, 59]. Binding affinity comparison of purified SHBG/ABP with digested protein fragments of prostate receptor also identified a conserved receptor-binding domain between amino acid residues 48-57 [41].

#### 2.1.1 Ablation of Androgen-Binding Region in Murine shbg Gene

Murine shbg gene (NT 096135) is 3816bp in length and encodes testicular ABP mRNA (U 8564, 1369bp in length. Exons 1-8 of murine ABP express testicular ABP proprotein composed of 403 amino acids, inclusive of signal peptide. Signal peptide cleavage in the endoplasmic reticulum during translation produces a mature protein comprising 373 amino acids [35, 49]. The mature protein undergoes post-translational modifications and is N-glycosylated at two positions. Glycosylation ensures a secretory role but is not a pre-requisite for steroid binding [57, 60]. The steroid-binding site of SHBG in each protomer is highly conserved in amino-terminal LG domain encoded by exons 2-5. Crystal structure of SHBG monomer, determined from N-terminal G domain polypeptide expressed in E Coli, revealed that DHT intercalates into a hydrophobic pocket between two anti-parallel β-sheets [61]. Ser42 in the amino-terminal LG domain of human SHBG is critical for binding steroids [62, 63]. Deletion of the androgen-binding region of shbg gene would affect the binding of iT/iD to ABP while retaining the androgen-independent functions of ABP [40]. Therefore, development of mice lacking the steroid-binding domain would be an effective strategy for studying the role of ABP in T retention, iT storage and spermatogenesis. Most importantly, retained glycosylation sites will not affect the secretion of the mutant protein. Southern blotting, immunohistochemistry, Western blotting and RIA will confirm the efficacy of ablation of shbg transcripts and ABP protein. The mutant model will also provide information about the independent role, if any, of ABP molecule in spermatogenesis. A systematic evaluation of iT targets in conditional ABPKO mice lacking T binding domain will reveal the effects of breakdown of the mechanism of iT retention and storage on spermatogenesis at adulthood.

### 2.2 Proposed Comparative Mechanism for Transport, Sequestration and Retention of Intratesticular T (iT) in Human and Rat Sertoli Cells (Fig. **1**):

Leydig cells secrete lipophilic hormone T in peripheral circulation [64, 65]. T ostensibly could interact with Sertoli cells through several pathways. Conventional mechanism of intravascular T action involving induction of Calcium influxes *via* non-genomic AR on Sertoli cells leading to CaM/CaMKIV/CREB activation [66-69]. Another mechanism would involve activation of intracellular signaling *via* SHBG-receptor (SHBGR) complex on plasma membrane of Sertoli cells [40]. Free T could enter Sertoli cells by diffusion but its lipophilic nature would limit intracellular bioavailability [61]. A more plausible pathway for T to enter Sertoli cells would be *via* endocytosis of vascular androgen-binding protein(s). The binding protein(s) would solubilize the lipophilic T and that would ensure bioavailability at nuclear AR. Indeed, sex steroid dependent tissues express transmembrane receptor megalin for endocytosis of plasmatic sex steroids. Sertoli cells do internalize sex steroids *via* megalin since Megalin receptor null mice have developmental defects like cryptorchid testis [38]. Sertoli cell aromatase and 5alpha-reductase enzymes aromatize or reduce iT to iE and iD, respectively. T/iD and iE act *via* nuclear AR and ER for gene expression. Germ cells express ER but not AR. ABP would transport iT to germ cells for aromatization to generate E2. E2 regulates several germ cell genes *via* EREs (demonstrated by CHIP assays), including repressed transcripts TPs and P (demonstrated by real-time PCR) in PPT/DPN-treated rats, that are eventually stored in the chromatoid body [15, 16, 33, 70]. It is tempting to speculate that germ cells may also endocytose SHBG/ABP *via* putative transmembrane receptors. Human germ cell lineages express a non-secretory variant of SHBG (aSHBG). ASHBG variant retains T binding function though the physiological relevance is not evident. ASHBG/ABP could serve as iT storage molecule in germ cells. It could also protect germ cells from excess iE exposure (akin to protection of developing fetus from overexposure to T).

## CONCLUSION

It is important to delineate the physiological mechanism(s) involved in sequestration and retention of iT and iD in mammalian Sertoli cells. Testosterone regulates expression of Sertoli cell genes involved in spermatogenesis. Testosterone is a lipophilic steroid hormone. The hydrophobic nature would render it insoluble in the hydrophilic cytosol and limit accessibility to nuclear AR. Most mammals’ express specific androgen-binding proteins for intravascular and intracellular retention of T. Androgen-binding proteins solubilize T/DHT. However, the mechanism of Sertoli cell iT storage awaits elucidation. Overexpression of ABP in transgenic mice led to upregulation of germ cell estrogenic mechanism. Therefore, development of conditional ABP null mice would be an inherently superior approach to delineate the mechanism of iT retention and storage in testis.

## Figures and Tables

**Fig. (1):**
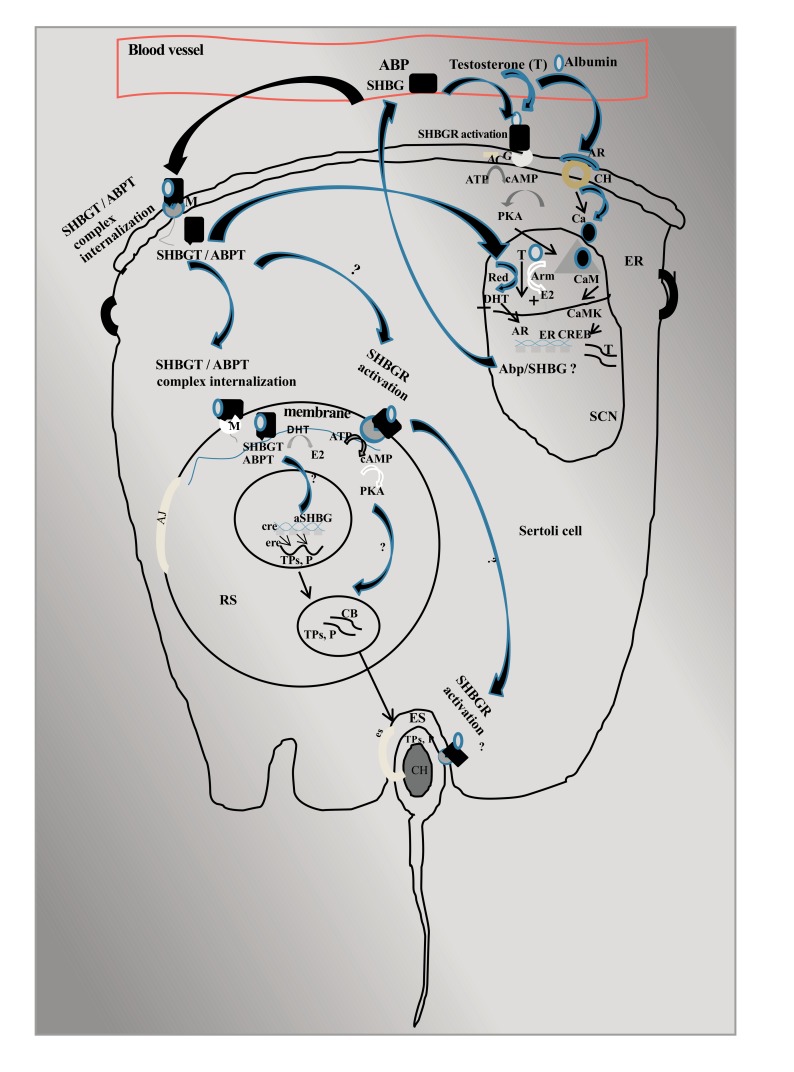
Schematic mechanism for androgen retention in rat Sertoli cells.

## LIST OF ABBREVIATIONS (FIG 1):

ABP: = Androgen binding protein
aSHBG: = Alternative transcript of SHBG
AC: = Adenyl cyclase
AJ: = Adhesion junctions
AR: = Androgen receptor
Arm: = Aromatase
Ca: = Calcium
CaM: = Calcium modulated protein
CamKIV: = Calmodulin kinaseIV
CB: = Chromatoid body
CH: = Condensed chromatin
cre: = cyclicAMP response element
CREB: = CyclicAMP response element-binding protein
DHT: = Dihydrotestosterone
E2: = Estradiol
ERE: = Estrogen response element
ER: = Endoplasmic reticulum
ER': = Estrogen receptor
ES: = Ectoplasmic specialization
FSH: = Follicle stimulating hormone
GC: = Germ cell
GCN: = Germ cell nucleus
M: = Megalin
P: = Protamine;
PKA: = Protein kinase A
Red: = 5alpha-reductase
SC: = Sertoli cell
SCN: = Sertoli cell nucleus
SHBG: = Sex hormone-binding globulin
SHBGR: = SHBG receptor
T: = Testosterone
TP: = Transition proteins
